# A Novel Deep-Trench Super-Junction SiC MOSFET with Improved Specific On-Resistance

**DOI:** 10.3390/mi15060684

**Published:** 2024-05-23

**Authors:** Rongyao Ma, Ruoyu Wang, Hao Fang, Ping Li, Longjie Zhao, Hao Wu, Zhiyong Huang, Jingyu Tao, Shengdong Hu

**Affiliations:** 1 School of Microelectronics and Communication Engineering, Chongqing University, Chongqing 400044, China; marongyao@126.com (R.M.);; 2 China Resources Microelectronics (Chongqing) Limited, Chongqing 401331, China; 3 Science and Technology on Analog Integrated Circuit Laboratory, Chongqing 401332, China

**Keywords:** silicon carbide, deep trench, super-junction, specific on-resistance

## Abstract

In this paper, a novel 4H-SiC deep-trench super-junction MOSFET (Metal-Oxide-Semiconductor Field-Effect Transistor) with a split-gate is proposed and theoretically verified by Sentaurus TCAD simulations. A deep trench filled with P-poly-Si combined with the P-SiC region leads to a charge balance effect. Instead of a full-SiC P region in conventional super-junction MOSFET, this new structure reduces the P region in a super-junction MOSFET, thus helping to lower the specific on-resistance. As a result, the figure of merit (*FoM*, *BV*^2^/*R_on,sp_*) of the proposed new structure is 642% and 39.65% higher than the C-MOS and the SJ-MOS, respectively.

## 1. Introduction

Silicon carbide (SiC), with the advantages of wide-band-gap semiconductors, is widely used in power MOSFET devices to obtain lower on-state resistance (*R_on_*), higher breakdown voltage (*BV*), and better frequency characteristics [1,2,3,4]. With the development of SiC MOSFETs and industry applications, requirements have been raised for SiC MOSFETs, and corresponding new structures have been proposed. Super-junctions are a good candidate device structure and have been introduced into SiC-based MOSFET devices [5,6,7,8]. In the charge balance theory of super-junction structures, this structure can break the material limit of the tradeoff between the breakdown voltage and the specific on-resistance (*R_on,sp_*) and achieve a lower *R_on,sp_* while keeping the *BV* in reasonable ranges [9,10,11].

A structure with an ultra-low specific on-resistance of 0.63 mΩ·cm^2^ at 1170 V has been successfully fabricated and published [12] and demonstrated the great potential of SiC super-junction (SJ) MOSFETs. At 3300 V, a *R_on,sp_* of 3.3 mΩ·cm^2^ has been reached as well [13]. Meanwhile, novel structures have been published to further improve device performance. A device formed with a high-k material offers a wider charge balance window than a novel SJ MOSFET [14,15] and, hence, reduces manufacturing error. Recently, deep-trench SJ MOSFETs with better short-circuit performance and electric field distribution have also been obtained [16,17].

Among all the studies on SiC SJ MOSFETs, the majority of device structures are based on planar gate MOSFETs, and only a few of them focus on trench-gate structures. Also, all of the structures use a single deep P-SiC in the drift region to provide charge balance effects in their devices. Although increasing the doped N concentration in the n-type drift area can reduce some amount of specific on-resistance, the occupied area limits the possibility of better on-resistance performance. Furthermore, it is difficult to produce SiC SJ MOSFETs with the traditional producing process for silicon MOSFETs because of the low diffusion rate in SiC, and the practicable process is much more complex and expensive.

In this paper, a novel deep-trench split-gate super-junction 4H-SiC MOSFET device structure is proposed and then theoretically verified with a Sentaurus TCAD simulation. The new structure shows a better performance in break-down voltage than a conventional trench MOS, which is comparable to a super-junction MOS, but it has better specific on-resistance than both structures, leading to a better *FoM.* Moreover, the new structure has a lower switching loss and eliminates the need for multiple epitaxial growth steps to form the P region.

## 2. Device Structure and Work Mechanism

**Figure 1:**
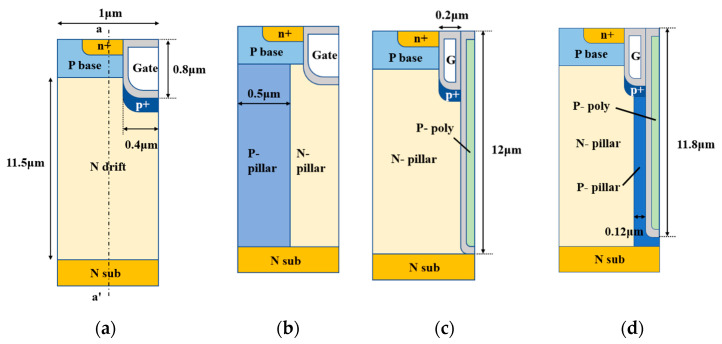
Cross-sectional view of (**a**) C-MOS, (**b**) SJ-MOS, (**c**) DTSJ-MOS, and (**d**) the new structure.

Figure 1a–d show cross-sectional views of a conventional trench MOSFET (C-MOS), a trench gate super-junction MOSFET (SJ-MOS), a deep-trench super-junction MOSFET (DTSJ-MOS), and the new device structure. The C-MOS and the SJ-MOS are conventional structures. All of the four structures are shown with the same cross-section areas.

The proposed structure has two trenches, a shallow one and a deep one. The C-MOS (a) and the SJ-MOS (b) have similar shallow gate trench structures with the same width. The gate trench is filled with n-type poly-Si and connected to a gate electrode. Since the shallow trench sizes of the C-MOS and SJ-MOS devices are the same, the gate width of the new structure (c) is smaller compared with the C-MOS and SJ-MOS, which reduces its reverse transfer capacitance (*C_rss_*), thus leading to a lower gate–drain charge (*Q_gd_*) and corresponding switching loss. The deep trench is filled with P-poly-silicon and connected to the source electrode. The deep trench is thinner than the shallow trench and can effectively induce a charge balance effect and form a super-junction-like structure. The deep trench is protected with a P-SiC region, which also helps to balance the charge with the N-pillar region. The oxide in the deep trench area provides a coupled charge to the charge balance as well. A conventional SJ-MOS uses half of the cell area as a P-pillar for the super-junction region, which not only narrows the current path but also causes complexity in the fabrication process. The new structure uses 0.1 μm wide P-poly-silicon and 0.25 μm wide P-SiC instead of a normal SiC pillar; it greatly widens the current path and reduces the *R_on,sp_* and complexity of the fabrication process. Lastly, the P+ region below the gate trench connected to the source electrode is used to protect the gate oxide from the high electric field. For a better understanding of the charge balance effect provided by the P-poly, a similar DTSJ-MOS (d) without a P-SiC region is also compared in this study. The doping concentration of the P-poly in the DTSJ-MOS is 8.7 × 10^17^ cm^−3^ compared with 2.5 × 10^17^ cm^−3^ in the new structure for the charge balance study. The main parameters of the structures are summarized in Table 1.

During device simulations, Sentaurus TCAD (Synopsys Inc., Sunnyvale, CA, USA) was used to reveal the electric characteristics. SRH, AUGER, and Okuto Crowwell were used as models to describe trap-assisted recombinations [18,19,20], non-radiative processes, and breakdown analyses, respectively. The simulations were taken under the same *R_on_* (1 mΩ) and threshold voltage (*V_th_*) (2 V) at a temperature of 300 K for all structures.

## 3. Simulation Results and Discussion

**Figure 2:**
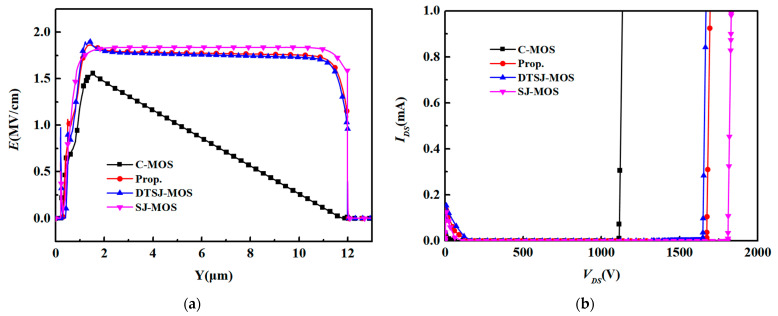
(**a**) Electric field distributions in the vertical direction. (**b**) The off-state characteristics.

Figure 2a shows the electric field distribution alone from a to a’ in the vertical direction, which is 0.5 μm from the cell’s left border. It can be seen that the electric field of the CMOS structure goes down when it closes to the drain electrode, while the other three structures keep it flat until it reaches the substrate, which indicates the charge balance effect exists in all of the three structures. Figure 2b compares the off-state characteristics of the structures. The *BV* values of the C-MOS, SJ-MOS, DTSJ-MOS, and new structure are 1113 V, 1809 V, 1690 V, and 1717 V, respectively. The high-doped P-poly in both the DTSJ-MOS and the new structure offers a charge balance effect together with the n-type SiC region, which forms a lateral electric field in the off-state and hence results in high breakdown voltage. Moreover, the P-SiC region surrounding the deep trench of the new structure further depletes the n-type pillar and is enhanced with the charge balance effect. However, the super-junction area is shorter than the SJ-MOS one, which causes a breakdown in the charge balance area of the p+ region under the shallow gate trench and leads to a lower *BV*.

**Figure 3:**
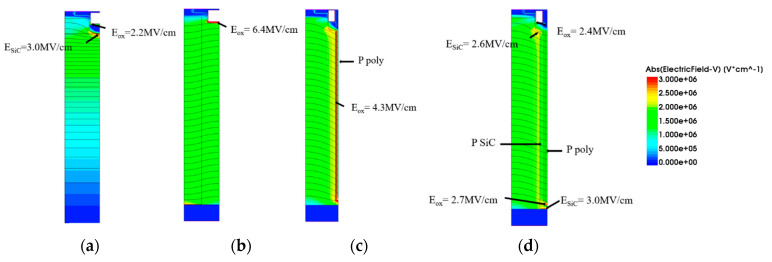
Off-state electric field contours of (**a**) C-MOS, (**b**) SJ-MOS, (**c**) DTSJ-MOS, and (**d**) proposed structure.

Figure 3 shows the electric field distributions of the C-MOS, SJ-MOS, DTSJ-MOS, and new structure in the off-state when avalanche breakdowns occur. Based on earlier research [21], a maximum oxide electric field (*E_MOX_*) of 2.7 MV/cm was estimated to maintain a lifetime of more than 10 years in a blocking state. The *E_MOX_* values of the four structures were 2.2 MV/cm, 6.4 MV/cm, 4.3 MV/cm, and 2.7 MV/cm, respectively. In the C-MOS and SJ-MOS, the *E_MOX_* appeared at the bottom corners of the gate trench; in the case of the DTSJ-MOS, it appears evenly at the side wall of the deep trench; and in the new structure, it occurs at the bottom corner of the deep trench. In the DTSJ-MOS and the new structure, the gate oxide is well protected by the P+ region, and the deep-trench oxide of the two structures is affected by the lateral electric fields because of the charge balance effect, which leads to a lower electric field. Since the deep trench is surrounded by a P-pillar at the bottom of the deep trench of the new structure, the charge balance is broken due to the enhanced electric field at the bottom. However, it also attracts the electric field, protecting the gate oxide from the high electric field peak. Meanwhile, the P-pillar helps the P+ region to distract the electric field, further protecting the gate trench. The SJ-MOS has the highest peak value of the electric field due to a lack of protection for the gate oxide, while the C-MOS and the new structure both have *E_MOX_* values under 2.7 MV/cm. Without the protection of the P-pillar, the peak electric field in the DTSJ-MOS is much higher than the new structure, and it is difficult to work with in real situations, whereas the new device has a compromise between the SJ-MOS and DTSJ-MOS. Therefore, the DTSJ-MOS will not be further discussed in the later discussions. Also, because of the charge balance effect provided by the super-junction structures, the electric potentials of the SJ-MOS, DTSJ-MOS, and new device are much more even than the C-MOS.

**Figure 4:**
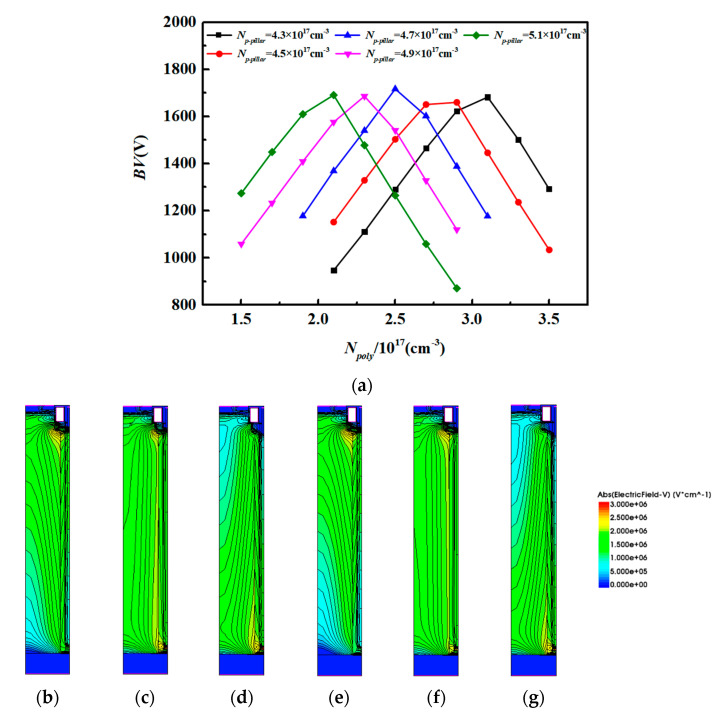

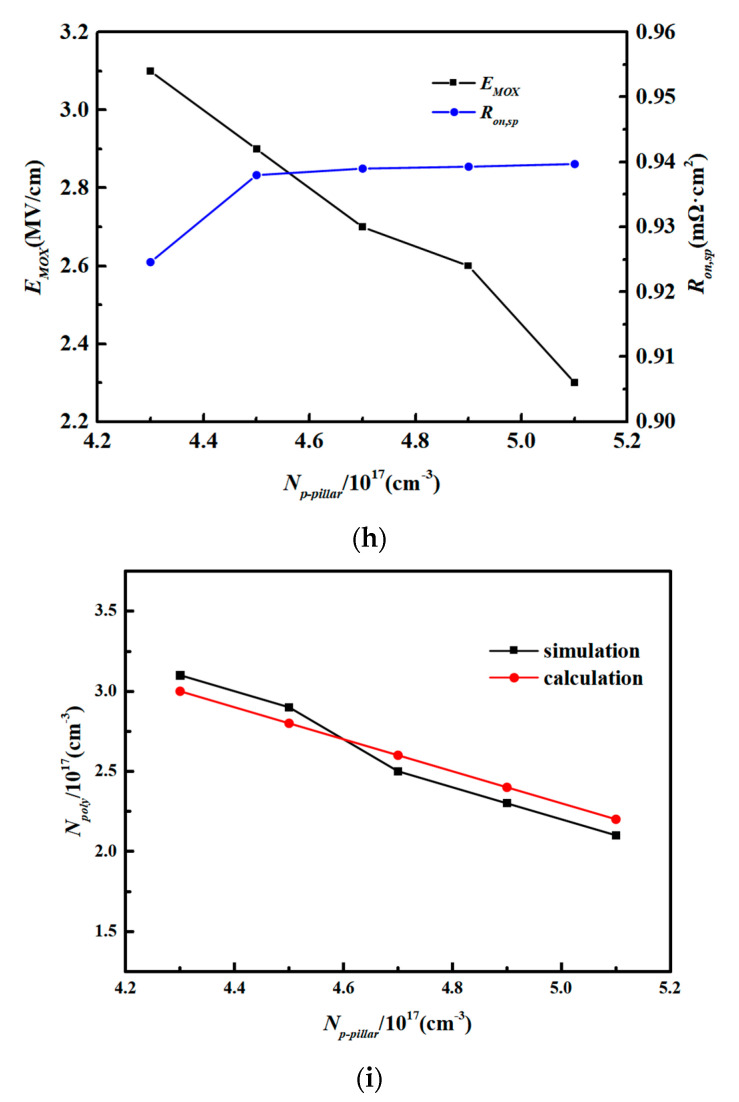
(**a**) Relationship between the *N_poly_*, *N_P-pillar,_* and *BV* of the new structure. (**b**) Electric field when *N_P-pillar_* = 4.3 × 10^17^ cm^−3^ and *N_poly_* = 2.5 × 10^17^ cm^−3^. (**c**) Electric field when *N_P-pillar_* = 4.3 × 10^17^ cm^−3^ and *N_poly_* = 3.1 × 10^17^ cm^−3^. (**d**) Electric field when *N_P-pillar_* = 4.3 × 10^17^ cm^−3^ and *N_poly_* = 3.5 × 10^17^ cm^−3^. (**e**) Electric field when *N_P-pillar_* = 4.7 × 10^17^ cm^−3^ and *N_poly_* = 2.1 × 10^17^ cm^−3^. (**f**) Electric field when *N_P-pillar_* = 4.7 × 10^17^ cm^−3^ and *N_poly_* = 2.5 × 10^17^ cm^−3^. (**g**) Electric field when *N_P-pillar_* = 4.7 × 10^17^ cm^−3^ and *N_poly_* = 3.1 × 10^17^ cm^−3^. (**h**) The *E_MOX_* of different *N_P-pillars_*. (**i**) Comparison of simulation results and calculation results of the relationship between *N_P-pillar_* and *N_poly_*.

Figure 4a shows the relationship between the *N_poly_*, *N_P-pillar,_* and *BV* of the new structure when the *N_N-pillar_* is set as 9.5 × 10^16^ cm^−3^. Because of the limitation in size control during the fabrication process, the widths of the deep trench and the P-pillar are set as 0.2 μm and 0.24 μm. For each *N_poly_*, there must be a specific *N_P-pillar_* to reach the best *BV*. With an increase or decrease in *N_poly_*, the *N_P-pillar_* must decrease or increase accordingly. The tradeoff between the *N_poly_* and *N_P-pillar_* follows the charge balance rule. For a conventional super-junction MOSFET, the charge balance effect can be described in the equation below:

(1) $$N_{N-pillar}\times W_{N-pillar}=N_{P-pillar}\times W_{P-pillar}$$

Here, *N_N-pillar_* and *N_P-pillar_* stand for the doping concentration of the *N-pillar* and the *P-pillar*, and *W_N-pillar_* and *W_P-pillar_* represent the widths of the *N-pillar* and *P-pillar*. However, the new device is more suitable for the revised equation, as follows:

(2) $$N_{N-pillar}\times W_{N-pillar}=N_{P-pillar}\times W_{P-pillar}+N_{P-poly}\times W_{P-poly}+Q_{c}$$

Here, the parameter *Q_c_* stands for the coupling charge, with an uncertain number around 1.3 × 10^13^ cm^−2^. The reason might be a coupling effect happening in the deep trench oxide.

Figure 4b–d show electric field distributions when the *N_poly_* changes from 2.5 × 10^17^ cm^−3^ to 3.5 × 10^17^ cm^−3^ while keeping the concentration of *N_P-pillar_* at 4.3 × 10^17^ cm^−3^. Figure 4e–g shows the electric field distributions when the *N_poly_* changes from 2.1 × 10^17^ cm^−3^ to 3.1 × 10^17^ cm^−3^ while keeping the concentration of *N_P-pillar_* at 4.7 × 10^17^ cm^−3^. The figures show that the effect of charge balance in the new structure is similar to the conventional super-junction MOSFET. The peak electric field occurs at the bottom of the deep trench, which is mainly affected by the distribution of *p*-type doping. Figure 4h shows the relationship between the *E_MOX_*, *R_on, sp,_* and *N_P-pillar_*. With the increasing *N_P-pillar_*, the field protection for the deep trench becomes stronger, and hence, the *E_MOX_* decreases. When the *N_P-pillar_* is lower than 4.7 × 10^17^ cm^−3^ (which means that the *N_poly_* is higher than 2.5 × 10^17^ cm^−3^), the *E_MOX_* will be over 2.7 MV/cm and becomes unacceptable. However, the *R_on,sp_* rises slowly with the *N_P-pillar_*, which indicates that the *N_P-pillar_* has little effect on *R_on,sp_*. Also, to make full use of *P-poly* and increase the manufacturing window of the *P-pillar*, the *N_poly_* should be as high as possible. As a result, we selected the values of *N_poly_* and *N_P-pillar_* to be 2.5 × 10^17^ cm^−3^ and 4.7 × 10^17^ cm^−3^, respectively.

Figure 4i shows a comparison of the simulation results and calculation results of the relationship between the *N_P-pillar_* and *N_poly_*. The values of the *N_P-pillar_* and *N_poly_* are chosen when the *BV* of the proposed structure reaches its maximum. As can be seen from the figure, the trends of the two curves are basically the same, which means that both the simulation results and calculation results prove that there is a charge balance effect in the proposed structure.

**Figure 5:**
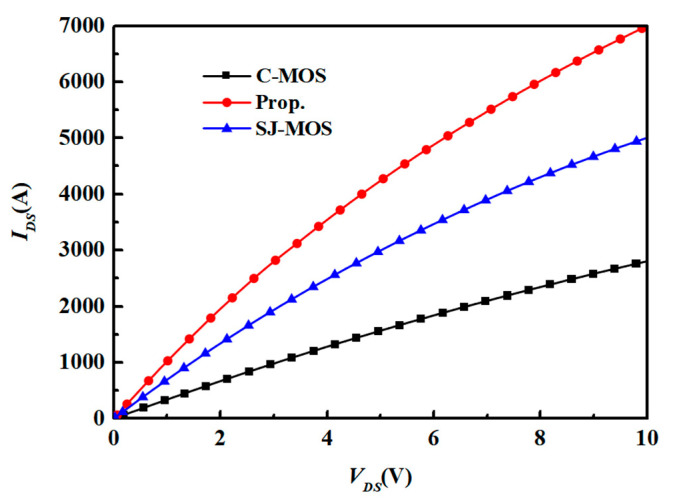
First quadrant I-V characteristics of the C-MOS, SJ-MOS, and new structure.

Figure 5 shows the first quadrant I–V characteristics of the three structures. The gate voltage (*V*_gs_) is 15 V. The *R_on,sp_* for the new device is 0.93 mΩ·cm^2^, which is about 67.93% lower than the 2.9 mΩ·cm^2^ of the C-MOS and 35.41% lower than the 1.44 mΩ·cm^2^ of the SJ-MOS. The doping concentration of the N-pillar for the SJ-MOS and new structure is 9.5 × 10^16^ cm^−3^ compared with 7.5 × 10^15^ cm^−3^ for the C-MOS. A lower concentration greatly increases the total electron current in the drift region and results in a significant reduction in *R_on,sp_*. At the same time, the new structure uses thin P-poly-silicon within a deep trench to replace the conventional P-SiC pillar in the SJ-MOS; the current path is further enlarged, which leads to a much lower *R_on,sp_*.

**Figure 6:**
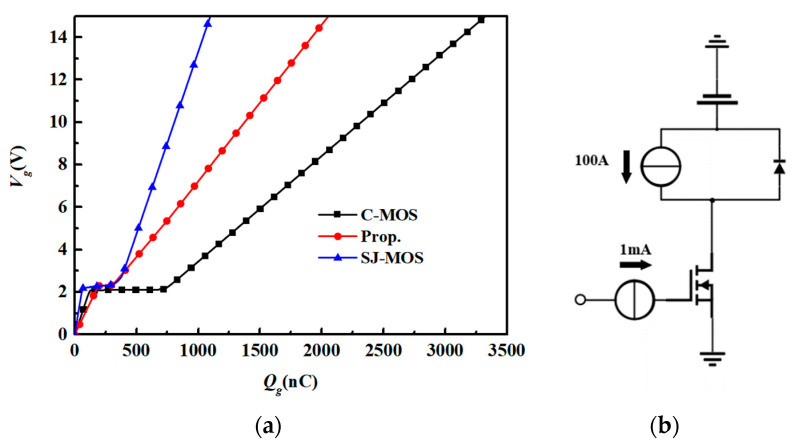
(**a**) Gate charge characteristics for the C-MOS, SJ-MOS, and new structure. (**b**) Test circuit.

Figure 6a,b show the gate charge curves of the devices and the simulation circuit. With the same *V_th_*, the height of the miller plateau of the three structures is the same. The new device takes the longest time to reach the Miller plateau, which indicates that it has the largest gate–source capacitance (*C_gs_*). This is because the deep trench connected to the source electrode enlarges the overlapping area of the gate and source electrode. The *Q_gd_* value of the new device is about 83.02% less than the one for the C-MOS and 62.73% less than the one for the SJ-MOS. In a super-junction structure, when the drain-source voltage (*V_ds_*) is not large enough to fully deplete the P-region, the gate–drain capacitance (*C_gd_*) is shielded by the junction capacitance formed through the N-pillar and P-pillar, so the *C_rss_* is small. At a high *V_ds_*, the P-pillar is fully depleted; the shielding effect is weakened; and, hence, the *C_rss_* will rise again. In the new device, the deep P-poly trench, together with the P-pillar, plays the role of shielding gate in the split-gate trench MOS and helps to shield the gate trench from the drain electrode. With the increasing *V_ds_*, the P-pillar is depleted first, and then, the *C_rss_* and output capacitance (*C_oss_*) rise, which limits the rising turn-on current under the high *V_ds_* and reduces the oscillation of the turn-on waveform. Meanwhile, the thin gate decreases the related area between the gate and the drain, and it further reduces the *C_gd_* and finally lowers the *Q_gd_*.

**Figure 7:**
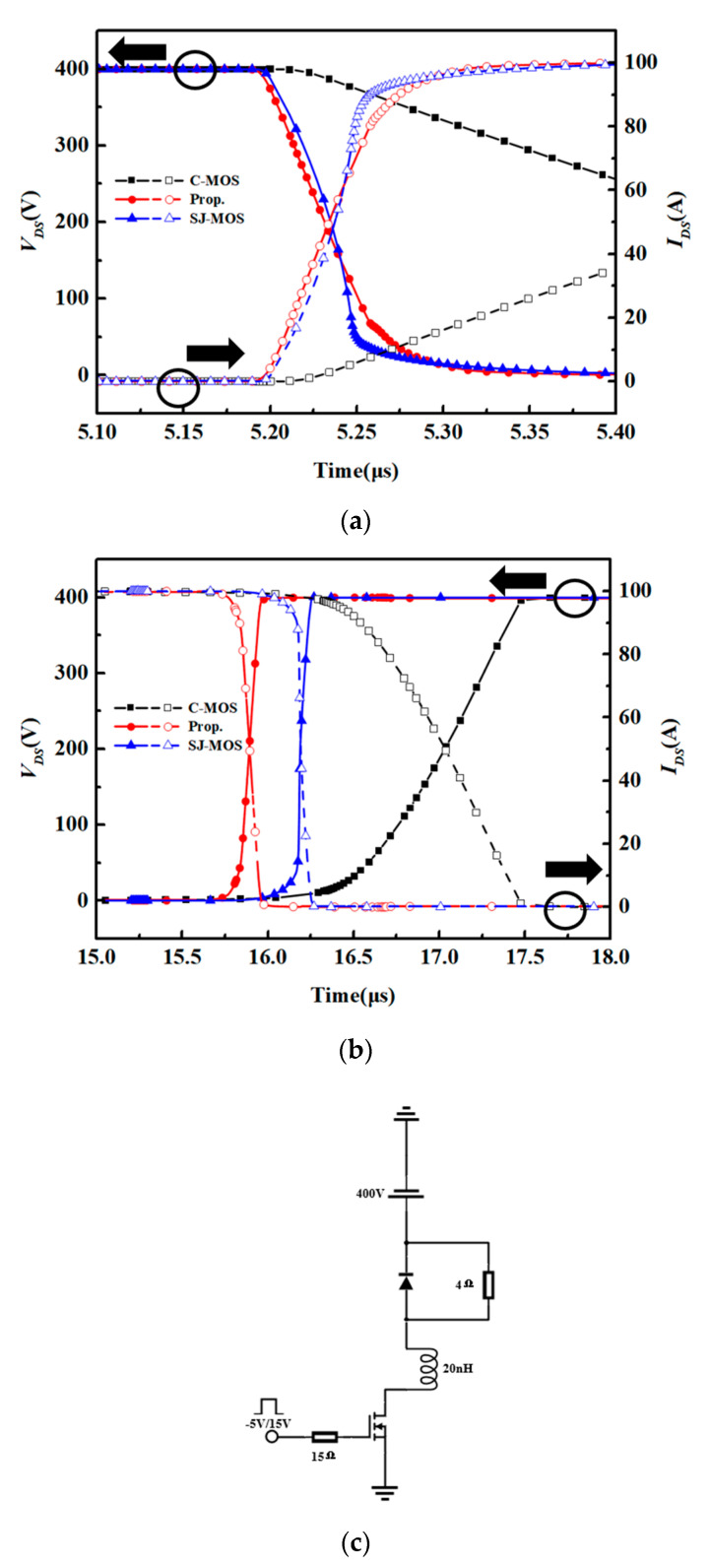
(**a**) The turn-on waveforms of the structures. (**b**) The turn-off waveforms of the structures. (**c**) Double pulse test circuits.

**Figure 8:**
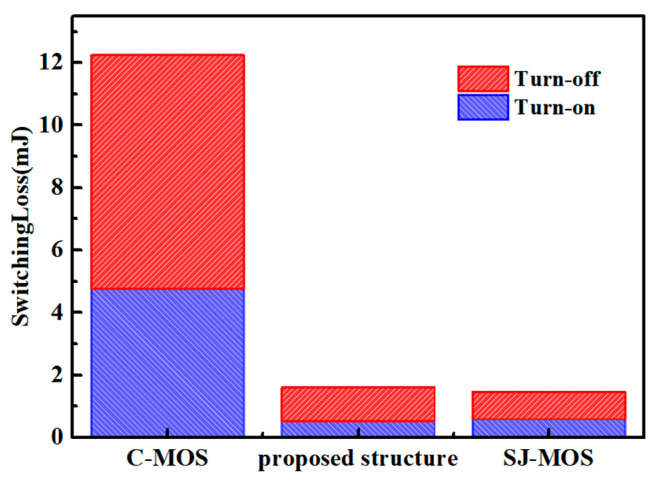
The switching-loss properties for various device structures.

The switching characteristics of the structures are shown in Figure 7. The simulation was applied with a typical testing circuit with an inductive load, as shown in Figure 7c. As can be seen, the turn-on and turn-off times of the new device are significantly shorter than the ones for the C-MOS and close to the values for the SJ-MOS due to the low *Q_gd_*. Because of the high switching speed, the d*I_ds_*/d*t* of the new structure is higher than the other structures. The switching loss of the different structures is shown in Figure 8. The turn-on and turn-off losses of the new device are 0.51 mJ and 1.09 mJ, respectively. In comparison, the turn-on and turn-off losses for the C-MOS are 4.76 mJ and 7.48 mJ. For the SJ-MOS, the values are 0.58 mJ and 0.88 mJ. As a result, the total switching loss of the new device is fair to the SJ-MOS and about 79.45% less than the one for the C-MOS. It is thus demonstrated that the new device has a superior switching character.

**Figure 9:**
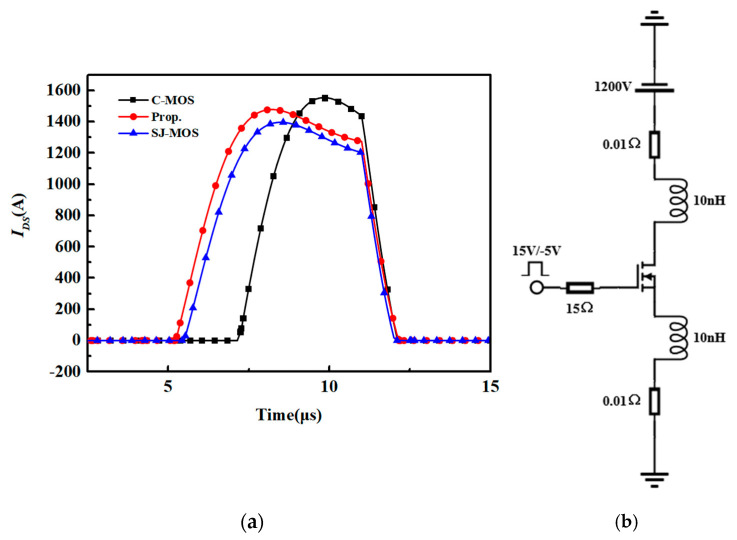
(**a**) The short-circuit waveforms for three different structures. (**b**) The short-circuit test circuits.

The short-circuit characteristics of the devices are shown in Figure 9. The *V_ds_* is set as 800 V, and the *V_gs_* is 15 V. The saturation current of the new device is 1480 A, which is higher than 1395 A for the SJ-MOS and lower than 1553 A for the C-MOS. The JFET area for the new structure is much wider than the SJ-MOS, and it weakens the current-limiting ability since the current path is greatly enlarged. However, both of the two super-junction structures show better short-circuit abilities than the C-MOS, and the P-pillar offers a pinch-off function during the short-circuit period. Considering the low *R_on,sp_* achieved by the new device, its short-circuit ability is acceptable. Table 2 summarizes the main electrical characteristics of the structures.

## 4. Proposed Fabrication Process

**Figure 10:**
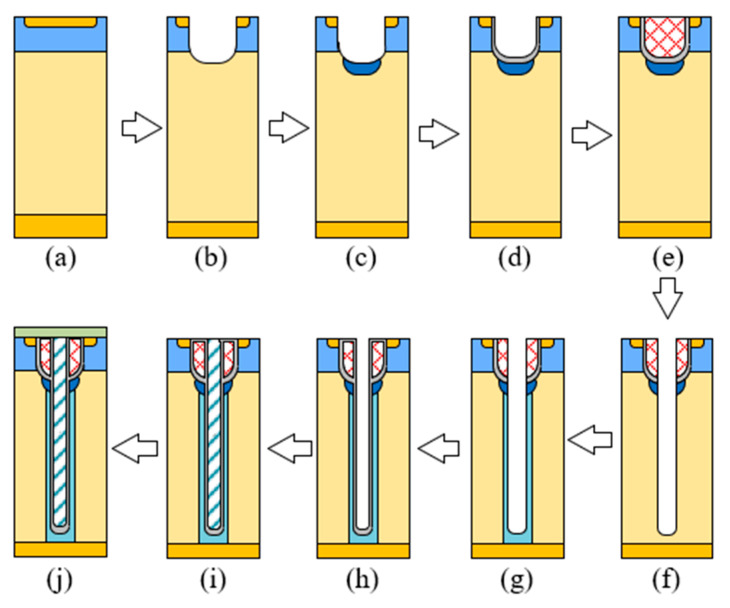
The key fabrication process: (**a**) Form a P well and N+ source. (**b**) Use ICP etching to form a shallow trench. (**c**) Form a P+ region through ion implantation. (**d**) Use thermal oxidation to form gate oxide. (**e**) Deposit gate poly-silicon. (**f**) Use ICP etching to form a deep trench. (**g**) Form a P-pillar through ion implantation. (**h**) Use thermal oxidation to form a deep trench oxide. (**i**) Fill the deep trench with P-poly. (**j**) Deposit the metal.

Regarding the feasibility of the new structure, a fabrication process is proposed in Figure 10. Compared with the SJ-MOS, the fabrication of the new structure is much easier to process because there is no need for a large p-SiC area. First, the N drift region is formed by epitaxial growth on a N+ substrate. Then, the P well and the N source area are made through ion implantation. Then, a shallow trench is fabricated by ICP (Inductively Coupled Plasma) etching, and the P+ protect area is formed by vertical implantation. After that, thermal oxidation is performed on the shallow trench. By filling it with n-type poly-silicon, a gate trench is formed. Then, we etch again to form a deep trench and implant to form a deep P-pillar. Thermal oxidation forms a deep trench oxide layer. Finally, the deep trench is filled with P-poly and connected to the source electrode through the metal layer, and the device is formed.

## 5. Conclusions

In this paper, a novel 4H-SiC-based deep-trench super-junction MOSFET with a split-gate is proposed, and the device’s properties are confirmed by using Sentaurus TCAD simulations. The novel structure shows a better tradeoff between the break-down voltage and the specific on-resistance. The new device can achieve an excellent *FoM* value, which is about 642% higher than the value for a C-MOS and 39.65% higher than the value for an SJ-MOS. Meanwhile, the deep trench in the new structure helps to reduce the *Q_gd_*. Finally, a feasible fabrication process with the advantage of a much easier process than the one for a SiC super-junction structure is proposed.

## Figures and Tables

**Table 1:** Main parameters used in the simulation.

Parameters	C-MOS	SJ-MOS	DTSJ-MOS	Prop.	Unit
Cell pitch (full cell)	2	2	2	2	μm
Depth of gate trench	0.8	0.8	0.8	0.8	μm
Width of gate trench (full cell)	0.8	0.8	0.2	0.2	μm
Depth of N drift	11.5	11.5	11.5	11.5	μm
Width of deep trench (full cell)	/	/	0.2	0.2	μm
Depth of deep trench	/	/	12	11.8	μm
Width of P-pillar (full cell)	/	1	/	0.24	μm
N drift doping	8 × 10^15^	9.5 × 10^16^	9.5 × 10^16^	9.5 × 10^16^	cm^−3^
P-pillar doping	/	9.5 × 10^16^	/	4.7 × 10^17^	cm^−3^
P-poly doping	/	/	8.7 × 10^17^	2.5 × 10^17^	cm^−3^
Thickness of gate trench (side)	40	40	40	40	nm
Thickness of gate trench (bottom)	80	80	80	80	nm
Thickness of deep trench (side)	/	/	50	50	nm
Thickness of gate trench (bottom)	/	/	100	100	nm

**Table 2:** Device characteristics comparison.

Parameters	C-MOS	SJ-MOS	Prop.	Unit
BV	1113	1809	1717	V
*R_on,sp_*	2.9	1.44	0.93	mΩ·cm^2^
*Q_gd_*	595	271	101	nC/cm^2^
BFOM (BV^2^/*R_on,sp_*)	427.2	2273	3170	MW/cm^2^
HF-FOM (*R_on,sp_* × *Q_gd_*)	1725.5	390.24	93.93	mΩ·nC
Switching Loss	12.24	1.46	1.60	mJ
*I_sat_*	1553	1395	1480	A

## Data Availability

The original contributions presented in the study are included in the article, further inquiries can be directed to the corresponding author.
